# Fertility rates and perinatal outcomes of adolescent pregnancies: a retrospective population-based study**^1^**

**DOI:** 10.1590/1518-8345.1820.2876

**Published:** 2017-04-06

**Authors:** Maria de Lourdes de Souza, Fiona Ann Lynn, Linda Johnston, Eduardo Cardoso Teixeira Tavares, Odaléa Maria Brüggemann, Lúcio José Botelho

**Affiliations:** 2 PhD, Professor, Universidade Federal de Santa Catarina, Florianópolis, SC, Brazil.; 3 PhD, Professor, School of Nursing & Midwifery, Queen's University, Belfast, Northern Ireland, United Kingdom.; 4 PhD, Professor, Lawrence S. Bloomberg Faculty of Nursing, University of Toronto, Toronto, CA, Canada.; 5 Physician, Hospital Baía Sul, Florianópolis, SC, Brazil.; 6 PhD, Associate Professor, Universidade Federal de Santa Catarina, Florianópolis, SC, Brazil.; 7 Doctoral student, Universidade Federal de Santa Catarina, Florianópolis, SC, Brazil. Associate Professor, Universidade Federal de Santa Catarina, Florianópolis, SC, Brazil.

**Keywords:** Adolescent, Pregnancy in Adolescence, Fertility, Prenatal Care, Maternal Health, Public Health

## Abstract

**Objective:**

analyze trends in fertility rates and associations with perinatal outcomes for adolescents in Santa Catarina, Brazil.

**Methods:**

a population-based study covering 2006 to 2013 was carried out to evaluate associations between perinatal outcomes and age groups, using odds ratios, and Chi-squared tests.

**Results:**

differences in the fertility rate among female adolescents across regions and time period were observed, ranging from 40.9 to 72.0 per 1,000 in mothers aged 15-19 years. Adolescents had fewer prenatal care appointments than mothers ≥20 years, and a higher proportion had no partner. Mothers aged 15-19 years were more likely to experience preterm birth (OR:1.1; CI:1.08-1.13; p<0.001), have an infant with low birthweight (OR:1.1; CI:1.10-1.15; p<0.001) and low Apgar score at 5 minutes (OR:1.4; CI:1.34-1.45; p<0.001) than mothers ≥20 years, with the odds for adverse outcomes greater for those aged 10-14 years.

**Conclusion:**

this study provides evidence of fertility rates among adolescents remaining higher in regions of social and economic deprivation. Adolescent mothers and their infants more likely to experience adverse perinatal outcomes. Nurses, public health practitioners, health and social care professionals and educators need to work collaboratively to better target strategies for adolescents at greater risk; to help reduce fertility rates and improve outcomes.

## Introduction

National fertility rates among adolescents are commonly used as an indicator for children and young people's health. While there has been a decline in the birth rate globally in recent years, pregnancy in adolescence remains a public health concern. The global birth rate, or age-specific fertility rate, for mothers aged 15 to 19 from 2007 to 2012 has been reported as 50 per 1,000. At national levels, higher rates are consistently reported for developing nations in sub-Saharan Africa; however, rates for Brazil are consistently higher than the global rates, with a rate of 68 in 1,000 reported in 2012, which is among the highest rates reported in Latin America and the Caribbean^1^. The World Health Organization (WHO) also reports that half of all births to adolescent mothers occur in just seven countries, Brazil being one of these^2^.

Adolescent pregnancy has been recognized as a major contributing factor to maternal and child mortality, morbidity and poverty^3^. Epidemiological studies indicate that adolescent pregnancies lead to a greater proportion of infants being born preterm and/or with low birth weight, which in turn have been associated with higher mortality rates^4^ ^-^ ^7^. Low socioeconomic conditions, risky lifestyle behaviors, poor adherence to prenatal care and biological immaturity have also been suggested as possible explanations for adverse obstetric and perinatal outcomes in this group^4^ ^-^ ^5^ ^,^ ^8^.

With the Millennium Development Goals 4 (reducing child mortality) and 5 (improving maternal health), public health strategies for tackling teenage pregnancy rates have been promoted in both developed and developing nations^9^ ^-^ ^10^. However, regional differences may persist and better targeting vulnerable populations may result in greater improvements. Consequently, evaluating the trends in fertility rates at a national and regional level is vital to comprehend any progress made and to indicate targets for the future.

The aim of this study is to understand the characteristics and health-related outcomes of live births to teenage mothers in comparison to adult mothers by analysing trends in age-specific fertility rates and associations with selected perinatal outcomes across one State in Brazil from 2006 to 2013 inclusive. The State of Santa Catarina has a population of approximately 6.7 million inhabitants. Regional figures state that 15.7% (n=1.04 million) of the population was aged 10-19 years in 2013, which is comparable to the national figure of 17.1% (n=34.29 million). The State of Santa Catarina consists of nine regions that differ in terms of social and economic development, with inherent health disparities and inequalities*. Public health policy makers at a regional level in Santa Catarina experience issues similar to other developing regions. As such, monitoring and understanding trends is an important step towards assessing the need for action, particularly in areas with limited resources.

## Methods

A population-based study covering the period from 2006 to 2013 was carried out. The research population included all live births across the State of Santa Catarina. Data were extracted from the Information System of Live Births (SINASC) database of the Ministry of Health, Brazil, through the DATASUS website, and from the State Health Department of Santa Catarina.

Age-specific fertility rates were calculated as a ratio, defined by the United Nations (UN)^*^ as the annual number of births to women of a specified age or age group per 1,000 women in that age group. Data for the State of Santa Catarina were collected for the years 2006-2013 inclusive. However, data by region were unavailable for the year 2013, due to the absence of regional data for the denominator. Hence, age-specific fertility rates at the regional level were calculated for the years 2006-2012.

Data on maternal characteristics included age (grouped as 10-14 years, 15-19 years, or ≥20 years of age), years of formal education (grouped as <8 or ≥8 years) and marital status, which was categorized as with partner (married or co-habiting) or without partner (single, widowed, separated or divorced). Pregnancy-related variables included the number of prenatal appointments attended (<7 or ≥7 appointments) and type of delivery (vaginal birth or caesarean section). Infant outcomes included gestational age at birth (<37 or ≥37 weeks), birth weight (<2500 grams or ≥2500 grams) and Apgar at 5 minutes, categorized as scores ≤7 or 8 to 10. The Apgar score establishes the clinical condition of all newborn infants immediately after delivery, regardless of the delivery mode, with a high score indicating good physical condition^8^. Aggregate data were collected on all live births by health region for 2006-2013. The nine regions in the State of Santa Catarina, as defined by the Health Department, are *Serra Catarinense* (Mountain Range)*, Extremo Oeste* (Great West)*, Meio Oeste* (Midwest)*, Foz do Rio Itajaí* (Itajaí River Mouth)*, Vale do Itajaí* (Itajaí Valley)*, Grande Florianópolis* (Greater Florianópolis)*, Sul* (South)*, Nordeste* (Northeast) and *Planalto Norte* (Northern Plateau). The UN Human Development Index (HDI) is a measure of the standard of living and takes into account education, life expectancy and population income. According to published HDI scores*, based on 2010 data, the regions of Santa Catarina with average lower HDI scores than the State score of 0.774 were *Extremo Oeste, Meio Oeste, Planalto Norte* and *Serra Catarinense*. The regions with higher HDI scores than the State included *Vale do Itajaí, Grande Florianópolis* and *Nordeste*.

### Data analysis

Population statistics relating to age-specific fertility rates among adolescents are most frequently reported for those aged 15 to 19 years. As such, data were analyzed by age groups 10 to14 years and 15 to 19 years. Age-specific fertility rates for female adolescents in the State of Santa Catarina were calculated for each year (2006-2013) to identify trends within this population. Odds Ratios (OR) and 95% Confidence Intervals (CI) were calculated to measure the level of association between specified age groups and perinatal outcomes. Chi-squared tests for statistical significance were also performed. Data were analyzed using electronic support. 

This study was developed in the context of Project TO 13075/2012 - FAPESC, Opinion No. 120.343, No. 169.110 in 2012. Ethical guidelines for research development were complied with in accordance with Resolution No. 466/2012 of the Brazilian Federal Health Department. This research did not involve direct recruitment and consent of human subjects, as secondary analysis was conducted on data collected from publicly available sources that contain anonymized data.

## Results

**Figure 1:**
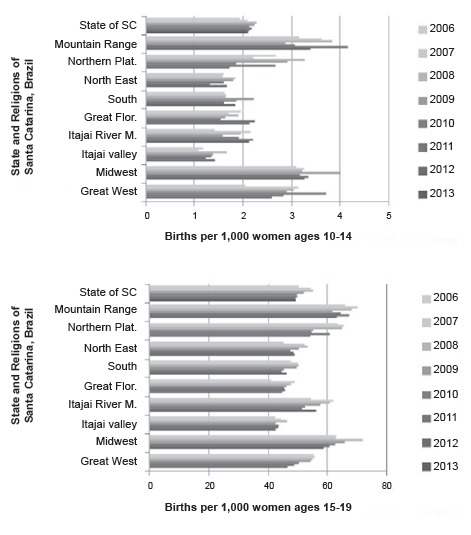
Trends in fertility rates among adolescents by State and region of Santa Catarina (SC), Brazil.

In Santa Catarina State, during the period 2006 to 2013, there were 115,559 live births to mothers aged 10 to 19 years, representing a proportion of nearly 17% of all live births (n=685,525). 3.8% (n=4,397) of births to adolescent mothers were in the youngest cohort aged 10 to 14 years. Across the State of Santa Catarina, the fertility rate to mothers aged 10-14 years ranged from 1.9 to 2.3 per 1,000 between 2006-2013 and from 49.1 to 55.0 per 1,000 female adolescents aged 15-19 years. In Figure 1, the trend in the fertility rate among female adolescents is illustrated in the state of Santa Catarina from 2006-2013 and by region, with data available for the period 2006-2012, indicating an overall steady decline. Birth rates to mothers' aged 10-19 remain highest in the regions, with an average lower standard of living (*Serra Catarinense, Planalto Norte, Meio Oeste* and *Extremo Oeste*). The regions with lower rates were the *Nordeste, Vale do Itajaí* and *Grande Florianópolis*, which represent largely urban areas that are more economically and socially developed, with higher average standards of living, as quantified by HDI scores**.

In Table 1, the years of formal education and marital status of the mothers are reported by age group for 2006 to 2013. With respect to education, 75% (n=3,277) of the adolescents aged 10-14 years had less than eight years of formal schooling; this proportion dropped to 38% (n=41,876) for those aged 15-19 years, as expected *a priori*. In the group of women aged ≥20 years, 72% (n=407,615) had eight or more years of education, with almost a third (28%; n=158,889) reporting less than eight years. As expected *a priori*, differences between groups in years of formal education and marital status were statistically significant (p<0.001). With regard to marital status, the groups 10-14 years and 15-19 years had a higher proportion of mothers living without a partner, 80.6% (n=3,545) and 66.6% (n=73,992), respectively. For women aged ≥20 years, this figure was 40.4% (n=230,534). 

**Table 1:** Level of education and marital status of the pregnant women by maternal age in Santa Catarina, Brazil, 2006-2013

Maternal characteristics		10-14 years	15-19 years	20 + years	P value*
		n^†^ (%)	n^†^ (%)	n^†^ (%)	
Education (years)					
	Less than 8 years	3,277 (74.5)	41,876 (37.7)	158,889 (27.9)	<0.001
	8 or more	1,089 (24.8)	68,614 (61.7)	407,615 (71.5)	
	Missing	31 (0.7)	672 (0.6)	3,462 (0.6)	
Marital status					
	Without partner	3,545 (80.6)	73,992 (66.6)	230,534 (40.4)	<0.001
	With partner	803 (18.3)	35,949 (32.3)	334,867 (58.8)	
	Missing	49 (1.1)	1,221 (1.1)	4,565 (0.8)	

*P value - Chi squared test for statistically significant differences between groups

† *n* - number of pregnant women

In Table 2, the pregnancy-related outcomes of prenatal appointments and type of delivery are reported, as well as infant outcomes of gestational age at birth, birth weight and Apgar score at five minutes for the three maternal age groups. Adolescent mothers were more likely to experience inadequate prenatal care, as defined by less than seven prenatal appointments, when compared to mothers aged ≥20 years, with the odds being greater for adolescents aged 10-14 years (OR: 2.40; 95% CI: 2.26-2.54; p value: <0.001) and those aged 15-19 years (OR: 1.72; 95% CI: 1.70-1.75; p value: <0.001). In relation to the type of delivery, the percentage of caesarean sections among those aged 10-14 years and 15-19 years was similar, with 43.2% (n=1,901) and 42.1% (n=46,751), respectively. This compares to a higher percentage among mothers aged ≥20 years (59.7%; n=340,537), with adolescent mothers statistically significantly less likely to have a caesarean section (p<0.001) than the older cohort, with statistical significance.

**Table 2:** Prenatal and perinatal outcomes for live births by maternal age group in Santa Catarina, Brazil, 2006-2013

Perinatal outcomes		10-14 years			15-19 years		20 + years
		n* (%)	OR^†^ (95% CI^§^ )		n* (%)	OR^†^ (95% CI^§)^	n* (%)
Prenatal care appointments							
	< 7	2,177 (49.5)	2.40^‡^		46,000(41.4)	1.72^‡^	165,892(29.1)
	≥ 7	2,194 (49.9)	(2.26-2.54)		64,497(58.0)	(1.70-1.75)	400,640 (70.3)
	Missing	26 (0.6)			665 (0.6)		3,434 (0.6)
Type of delivery							
	Caesarean section	1,901 (43.2)	0.51^‡^		46,751 (42.1)	0.49^‡^	340,537 (59.7)
	Vaginal birth	2,492 (56.7)	(0.48-0.55)		64,336 (57.9)	(0.48-0.50)	229,098 (40.2)
	Missing	4 (0.1)			75 (<0.1)		331 (0.1)
Length of gestation							
	< 37 weeks	598 (13.6)	1.71^‡^		10,266 (9.2)	1.10^‡^	48,198 (8.5)
	≥ 37 weeks	3,776 (85.9)	(1.57-1.86)		100,341 (90.3)	(1.08-1.13)	519,477 (91.1)
	Missing	23 (0.5)			555 (0.5)		2,291 (0.4)
Birth weight							
	<2500 grams	504 (11.5)	1.53^‡^		9,666 (8.7)	1.13^‡^ (1.10-1.15)	44,436 (7.8)
	≥2500 grams	3,893 (88.5)	(1.40-1.68)		101,486 (91.3)		525,479 (92.2)
	Missing	-			10 (<0.1)		51 (<0.1)
Apgar at 5 min							
	≤7	167 (3.8)	1.82^‡^		3,264 (2.9)	1.39^‡^	12,129 (2.1)
	8 to 10	4,211 (95.8)	(1.56-2.13)		107,692 (96.9)	(1.34-1.45)	556,731 (97.7)
	Missing	19 (0.4)			206 (0.2)		1,106 (0.2)

* n - number of pregnant women

† OR - Odds ratio with age group of 20 years or more as reference

‡ p <0.001 (P value - Chi-squared test for statistically significant differences between groups)

§ CI - Confidence Intervals

The number of preterm births (<37 weeks' gestation) was higher in the 10-14 years age group (13.6%; n=598), when compared to the other two maternal age groups: 15-19 years old (9.2%; n=10,266) and ≥20 years (8.5%; n=48,198). An odds ratio (OR) of 1.71 (95% CI: 1.57-1.86; p value: <0.001) confirms that those aged 10-14 years had a significantly higher chance of a preterm birth compared to women ≥20 years of age. This association continued for those aged 15-19 years (OR: 1.1; 95% CI: 1.08-1.13; p value: <0.001). With regard to the delivery of a low birth weight infant, pregnant women aged 10-14 years were more likely to have an infant weighing <2500g at birth (OR: 1.53; 95% CI: 1.40-1.68; p value: <0.001) compared to women ≥20 years. Although the odds were reduced for women aged 15-19 years, they remained statistically significant (OR: 1.13; 95% CI: 1.10-1.15; p value: <0.001). In relation to the Apgar score at 5 minutes, which is an indicator of the infant's physical condition; mothers aged 10-14 years were 1.82 times more likely to have an infant with an Apgar score of 7 or less (95% CI: 1.56-2.13; p value: <0.001); while mothers aged 15-19 years were 1.39 times more likely (95% CI: 1.34-1.45; p value: <0.001) when compared to infants born to mothers aged ≥20 years. When infant Apgar scores at 5 minutes were compared between adolescents aged 10-14 years and those aged 15-19 years, no statistically significant differences were observed (p=0.07).

## Discussion

The decline in the fertility rate among adolescents in the State of Santa Catarina is in line with the overall rate in Brazil, other developing nations and with the global trend reported by the WHO. In a comparative study of 29 countries in Africa, Asia, Latin America and the Middle East between 2010 and 2011, the average proportion of pregnancies to adolescents was 25.9%^11^. While Brazil and the State of Santa Catarina are below this average; they remain above average when compared to more developed countries^12^. With respect to fertility rate trends among adolescents across regions in the State of Santa Catarina, differences were observed. Regions with a lower standard of living continue to report higher fertility rates to adolescent mothers. These regions are typically characterized as experiencing more economic deprivation, with greater health, social and educational disparities. Adolescence is a stage of development that is greatly influenced by local cultural, social and economic conditions, alongside biological changes, psychological effects, lifestyle behaviors and social and cultural values^6^. The high number of adolescent pregnancies has previously been attributed to a failure of public health policies in the prevention of unplanned pregnancies and in sexual and reproductive health education reaching young people^13^. Many adolescents are deterred from engaging in the preference, prevention and promotion approach primary health care systems approach. As such, greater effort is needed in targeting appropriate strategies and effective interventions to adolescents in regions that are falling behind the State and/or Brazilian average^14^.

Complications in teenage pregnancies have previously been associated with adverse social conditions, low levels of education, marital status, lack of family support and, above all, inadequate prenatal care^13^. The findings from this paper support this evidence. Effective prenatal care is able to detect infectious diseases that have the potential for vertical transmission and a range of adverse conditions that may impair the health of the mother and fetus, such as malaria, HIV, rubella, syphilis and hepatitis. Above all, it is a simple task during a prenatal visit to check maternal and fetal vital signs, estimate gestational risks and assist in preventing adverse outcomes^15^. Prenatal care is a valuable tool for monitoring the health of the mother, as well as monitoring the on-going development of the fetus, and has been associated with lower rates of preterm birth and a reduction in infants of low birth weight^13^. Prenatal care for all adolescents therefore needs to commence early in gestation to ensure that early identification of risk takes place and that any follow-up actions required are taken^16^.

In this population-based study, the incidence of preterm birth (<37 gestation weeks) was higher among mothers aged 10 to 14 year olds than those aged 15 to 19 years and 20 years old or more, which is similar to figures reported in developing countries^12^. It should also be noted that the incidence of maternal near miss in childbirth and postpartum hospital in Brazil among adolescents aged 10-14 years has previously been reported as 15.7 per 1,000 live births and 9.8 per 1,000 live births for those aged 15-19 years; in the 20-34 years age group, this figure equals 9.4 per 1,000 live births^17^ ^). C^onsidering all teenagers as a single homogenous cohort creates a barrier for consensus on the issue of preventing maternal near miss^4^ ^-^ ^5^ ^,^ ^18^. The smaller cohort of mothers aged 10-14 years require personalized care in the intrapartum and postpartum period if improvements are to be observed. 

The data obtained on the birth weight of live births that occurred in Santa Catarina confirm previous findings in the literature on the relationship between maternal age and birth weight of infants, particularly highlighting the association between adolescent pregnancy and low infant birth weight. Previous studies have reported an average prevalence of low infant birth weight corresponding to 12% for all adolescent groups assessed (<16 years, 16-17 years and 18-19 years old)^13^ ^,^ ^19^, placing the State of Santa Catarina in a slightly better context than most low- and middle-income countries. Nevertheless, the birth weight of infants in developed and developing countries remains a major factor affecting neonatal morbidity and mortality, and is an important indicator of public health^6^ ^,^ ^8^. Likewise, nutrition and lifestyle habits post-conception (use of alcohol, tobacco, drug misuse, exposure to infections, such as malaria and HIV) also influence fetal growth and development, as well as the duration of pregnancy^16^. Women with unfavorable socioeconomic conditions often have infants with low birth weight, which in turn has been linked to poor nutrition and unhealthy lifestyle habits^5^ ^,^ ^16^.

Low birth weight may result in a risk to the survival and development of the infant. In addition, children with teenage mothers are in greater need of a social support network, which is not always available. Thus, the growth and development of these children, with follow-up health checks through attendance at well-baby clinics, is paramount. This requires nurses to take care of the adolescent mother/child dyad. The health care network has to be prepared to offer adequate support to those clients of greater vulnerability, both during attendance at the health services and at home. Adolescents are women in the process of discovering their sexuality and are not always prepared for motherhood^10^ ^,^ ^13^ ^-^ ^14^ ^,^ ^16^.

With regard to delivery, the preferred mode is vaginal, as long as there are no medical indications for a caesarean section, which may include two or more previous caesareans, fetal macrosomia or shoulder dystocia^20^. This study found that the prevalence of caesarean sections in pregnant women younger than 20 years old was lower than those aged 20 years or more (42% vs. 60%), with significantly lower odds. Prenatal care may be a mediating factor in this, with adolescents receiving fewer prenatal care appointments and, therefore, less exposure to the introduction of planned obstetric intervention prior to labor and delivery. Similar findings have been reported across Brazil, while lower figures of less than 30% have been reported among other low- and middle-income countries^11^. The figures for the prevalence of caesarean sections reported within this study, across all age groups, exceed recommendations from the WHO to limit caesarean sections to between 10-15% of all births^21^. New regulations are being enforced in 2015 in Brazil^22^, in an attempt to reduce the universally high rate of caesarean sections, by ensuring that pregnant women are informed of the risks, with the ultimate aim of reducing rates and creating a shift in the culture to informed choice and decision making by mothers.

In the early assessment of infant health, the Apgar score is an important tool to monitor the physical condition of newborns. According to the findings in this study, an Apgar score <7 occurred in 3% of births to adolescent mothers, which is similar to national data. A cohort study recently conducted in Brazil reported significant associations between neonatal mortality and birth weight, gestational age at birth, a low Apgar score at 5 minutes, the use of mechanical ventilation and congenital malformations^23^. However, the use of the Apgar score in preterm infants has been reported to be inconsistent and any assumptions with respect to the predictive value of the Apgar score for infant mortality should also take into account a range of other factors, including social and economic conditions^24^. Nevertheless, previous research aimed at assessing causal links between a range of maternal and pregnancy-related variables and infant Apgar scores reported associations between the frequency of prenatal care and an Apgar score of less than seven^13^. The authors provided evidence that appropriate prenatal care halves the risk of an unsatisfactory Apgar score^13^. With adolescent mothers in this population-based study being more likely to experience inadequate prenatal care, preterm birth, having an infant of low birth weight and a low Apgar score, it is essential that health services be more easily accessible and supportive of this vulnerable group. One such initiative is the CERCA study, currently conducted across three Latin American cities, which aims to develop, implement and evaluate a community-embedded reproductive health care program for adolescents. This innovative study has the potential to improve outcomes for adolescents through a targeted sexual and reproductive health program^25^.

Reproductive health care requires acknowledgement of the complexities involved in adolescent pregnancy. It needs to take into consideration the social and cultural factors that may contribute to its occurrence. The adolescents may view pregnancy as an opportunity to acquire the desired family autonomy. Aside from this, the adolescent may not recognize the pregnancy nor many of the associated risks or social losses they may experience in becoming a mother^18^ ^,^ ^26^.

In terms of study limitations, it should be noted that in calculating the fertility rates among adolescents aged 10-14 years, the denominator data related to all female adolescents aged 10-14 years in the State and each respective region. Consequently, the rates observed are likely to be an underestimation of the true fertility rate in this cohort. It would be more appropriate to employ an estimate of exposure to childbearing by females aged 10-14 years as the denominator. However, these data were unavailable. The reader should also reflect on the issue of reporting fertility rates among adolescents, instead of the rate of teenage pregnancy. This figure would undoubtedly be higher, as it would include not only all pregnancies that resulted in a live birth, but also those that resulted in a spontaneous miscarriage, termination of pregnancy or stillbirth. Understanding any disparities across regions in relation to the differences between pregnancy rates and fertility rates is difficult without the routine collection and dissemination of these data. Nevertheless, fertility rates are useful as a proxy measure at a local level to obtain a better understanding of trends, in order to target effective interventions and evaluate their efficacy.

## Conclusions

The findings from this current study raise awareness for the continued observation and on-going analysis of fertility rates among adolescent mothers. There is a clear need for greater attention to adolescent mothers in health care services, in order to assist them and their infants, particularly in regions identified as having higher rates of teenage pregnancy. Adapting current public health strategies to target adolescents living in regions with a lower standard of living may ensure that the disparities observed are minimized, with the provision of effective interventions targeting this vulnerable group. In addition, a network of specialized support and care to pregnant adolescents, which incorporates reproductive, prenatal, psychological and social support and care, may contribute to the reduction of obstetric and fetal risks.
